# A neurocomputational variable on welfare tradeoffs explains the function and form of cyberaggression

**DOI:** 10.3389/fnbeh.2023.1034564

**Published:** 2023-07-27

**Authors:** Jinguang Zhang

**Affiliations:** ^1^School of Journalism and Communication, Sun Yat-sen University, Guangzhou, China; ^2^Center for Big Data and Public Communication, Sun Yat-sen University, Guangzhou, China

**Keywords:** cyberaggression, welfare tradeoff ratio, anger, hatred, evolutionary psychology

## 1. Introduction

Cyberaggression is the intentional infliction of harm through information communication technologies (e.g., the Internet) (Smith et al., 2012). It is prevalent around the globe (Barlett et al., 2021), and causes in victims serious consequences including anxiety, depression, and suicide (Martínez-Monteagudo et al., 2020). To reduce the impact of cyberaggression, a critical first step would be to understand its why and how it occurs.

Prior research has provided much insight into the function and mechanism of cyberaggression. Regarding function, Runions et al. (2017) characterized cyberaggression along the dimensions of affective valence and levels of self-control and argued that cyberaggression may be carried out for revenge or reward, in either a calculated manner or impulsively. Lapierre and Dane (2020) argued that proactive cyberaggression facilitates intrasexual competition and dominance contests. Regarding mechanism, Kowalski et al.'s (2014) meta-analysis identified 10 risk factors, including being previously victimized, moral disengagement, anger, and narcissism.

In this opinion piece, we extend this line of work by developing a computational analysis of cyberaggression. Computational theories explain an information-processing device (e.g., the brain) by specifying (1) the problem that the device is designed to solve and (2) the mechanisms that must be in place to solve the problem (Marr, 1982; Cosmides and Tooby, 1995). As we aim to show in this article, our analysis would not only be able to integrate prior findings on the function and mechanism of cyberaggression but would also explain a previously overlooked aspect of cyberaggression, namely, the tactics it commonly comprises.

## 2. The welfare tradeoff ratio, anger and hatred

Humans as a highly social species often act in ways that negatively affect other's welfare relative to their own (i.e., things that however indirectly increase their fitness, or success in gene replication; Aktipis et al., 2018). For example, by taking the last vacant seat on a packed bus, one deprives another person of their opportunity to rest up and accomplish something productive later. People have thus been faced with the adaptive problem of deciding on how much they are willing to trade others' welfare for their own and their own for others'. Prior research suggests that the human brain contains a neurocomputational variable called the welfare-tradeoff ratio (WTR) that facilitates this decision-making process (Delton and Robertson, 2016).

Consider a behavior that would benefit Person A by *b* units of fitness (*b*_Person A_) while cost the focal individual (“you”) by *c* units of fitness (*c*_you_). Person A would perform the behavior when:


$$\begin{array}{l} b_{\text{Person A}}>WTR_{\text{Person A},\text{ you}}\times \;c_{\text{you}} \end{array}$$

In this inequality, WTR_Person A, you_ represents how much Person A values your welfare relative to their own, with the value of 1 indicating the person values their and your welfare equally. When WTR_Person A, you_ increases from 1, Person A would value your welfare relative to their own increasingly more, and when WTR_Person A, you_ decreases from 1, they would value your welfare less and less. For instance, a behavior would benefit Person A by 2 units of fitness and cost you by 3. Person A would not perform this behavior if they value you (with, e.g., a WTR_Person A, you_ = 1.5) but would if they do not value you (with, e.g., a WTR_Person A, you_ = 0.5). Recent research found that the mental computation of WTR bears the hallmarks of being a psychological adaptation (e.g., efficiency, economy, and precision) and is unlikely a heuristic (Delton et al., 2023).

From the perspective of evolutionary psychology, emotions are superordinate psychological adaptations that coordinate lower levels of mechanisms to solve complex adaptive problems (Sznycer et al., 2021). The computation of WTR is one such mechanism that many emotions incorporate. For example, the recalibration theory of anger (Sell et al., 2017) posits that a low perceived WTR_Person A, you_ informs you that Person A is not valuing you and causes you to be angry. This feeling motivates “loud” behaviors (e.g., aggressive postures, heated arguments) aiming to up-regulate WTR_Person A, you_ and salvage a cooperative relationship (between, e.g., two friends).

However, when WTR_Person A, you_ drops below zero, Person A's welfare and yours become negatively correlated (e.g., both of you desire the same job position), making Person A “toxic” to you (Sell et al., 2023). Because Person A thrives at your expense, recalibrating WTR_Person A, you_ is difficult if not impossible (think about Voldemort and Harry Potter). Hatred as a psychological adaptation solves this problem by (1) setting WTR_you, Person A_ at below zero and (2) motivating behaviors that would cost-effectively neutralize Person A's negative impact on your fitness. These behaviors include (1) predatory-style (e.g., surreptitious) aggression aiming to kill and (2) information warfare aiming to hurt one's reputation. The former tactic would help physically—whereas the latter would help socially—remove the toxic person from the hateful person's environment. Either way, the goal is to undermine the toxic person's ability to further impose costs on the hateful person.

## 3. A neutralization hypothesis of cyberaggression

The neutralization theory of hatred provides an integrative account of cyberaggression. First, hate speech is commonly observed on social media (Castaño-Pulgarín et al., 2021; Walther, 2022), suggesting that the emotion of hatred underlies many hurtful remarks people make on each other in cyberspace. Second, to the extent that cyberaggression is primarily hatred-based, it is directed at a toxic person and, by reducing the person's toxicity, would help increase the hateful person's fitness. That is likely why cyberaggression can be vindictive and rewarding at the same time (i.e., “revenge is sweet”) (Runions et al., 2017). Third, cyberaggression facilitates intrasexual competition and dominance contests (Lapierre and Dane, 2020) likely because both activities are examples where two persons' welfare is negatively correlated (e.g., jockeying for the same romantic partner or the only spot at the top of social hierarchy).

Fourth, prior research found that prior victimization, moral disengagement, anger, and narcissism positively predict the intent to cyberaggress (Kowalski et al., 2014). Of those predictors, being victimized before would likely make the motive of avenging chronically accessible to the victim and thus set their WTR toward others at values lower than people who have not been victimized before. Moral disengagement licenses harming others by (among other strategies) distorting consequences, displace responsibility, and dehumanizing the target. Anger positively predicts cyberaggression likely because it is closely related to hatred. Finally, more narcissistic people tend to have stronger senses of entitlement (Freis and Hansen-Brown, 2021) and are thus more likely to perceive infringements on their welfare, rendering a person who is otherwise neutral to their fitness subjectively toxic.

### 3.1. The form of cyberaggression

Our neutralization hypothesis of cyberaggression also explains why cyberaggression comprises the tactics that it does. By content-analyzing 29 published scales (Chun et al., 2020), we identified seven common tactics of cyberaggression (ordered by how frequently they appeared in the scales): (1) issuing insults and threats (96.6%), (2) public humiliation (e.g., posting embarrassing photos of someone; 72.4%), (3) spreading rumors (69.0%), (4) publicizing someone's dark secrets (69.0%), (5) social exclusion (58.6%), (6) impersonation (e.g., pretending to someone to post incriminating messages; 55.2%), and (7) sexual harassment (e.g., sending someone nude pictures; 44.8%). While those percentages indicate no current consensus on how to measure cyberaggression, there is perhaps a good reason to why people tend to use certain tactics more often than others when cyberaggressing.

Specifically, if cyberaggression is for decreasing someone's ability to impose costs, people should generally adopt tactics that would achieve that goal *cost-effectively* (Sell et al., 2023). Under this view, issuing insults has the highest percentage likely because executing this tactic entails the lowest amount of cost—one only needs to know the target's email or social media address—but can be highly effective in inflicting harm (Martínez-Monteagudo et al., 2020). In comparison, posting embarrassing photos (Tactic 2) and publicizing someone's secrets (Tactic 4) requires getting hold of something from the target, rumors (Tactic 3) may be falsified, and social exclusion (Tactic 5) needs coordination. As for Tactic 6, effective impersonation requires access to the target's online accounts, and unauthorized access is illegal. In other words, people are less likely to adopt Tactics 2 to 5 for cyberaggression likely because those tactics are increasingly costly to use and/or less and less effective to hurt the target.

### 3.2. The exceptional case of sexual harassment?

We condemn all forms of sexual harassment. However, if cyberaggression is for impairing a toxic person's ability to impose costs, sexual harassment (e.g., making unwanted sexual advances and requests for sexual favors) (The U.S. Equal Employment Opportunity Commission, 2023) appears the least frequently in extant scales of cyberaggression likely because it is the least suitable for that purpose. Inappropriate physical contact unlikely kills, and requests for sexual favors are often made with promises of benefit delivery (e.g., job promotion).

Sexual harassment *does* cause stress and fear in victims (Fitzgerald and Cortina, 2018) and may be weaponized as a means to harm. However, it does not follow that the psychological system producing sexual harassment is designed to harm. Inferring cause from outcome is a logical fallacy (Aktipis and Kurzban, 2005), just as paperweights may be used to kill but are not designed to kill. In fact, it has been argued that short-term mating motivates sexual harassment such that sexual harassment signals a perpetrator's—and probes the target's—interest in engaging in a sexual relationship (Jonason et al., 2012; Bendixen and Kennair, 2017).

Supporting this hypothesis, prior research found that unrestricted sociosexuality (e.g., “sex without love is OK”) but not hostile sexism positively and significantly predicted both men and women sexually harassing members of the opposite sex (Bendixen and Kennair, 2017; Zapata-Calvente et al., 2019). This finding provides no evidence that perpetrators hate the person they harass. Rather, it suggests that the perpetrator perceives the presence of the target as an opportunity to increase the perpetrator's reproduction. If this is the case, the perpetrator should hold a positive instead of a negative WTR toward the victim. This analysis suggests that sexual harassment may better be considered a different category of cyberaggression than the other six tactics mentioned earlier.

### 3.3. Predictions and ways to test them

**Figure 1:**
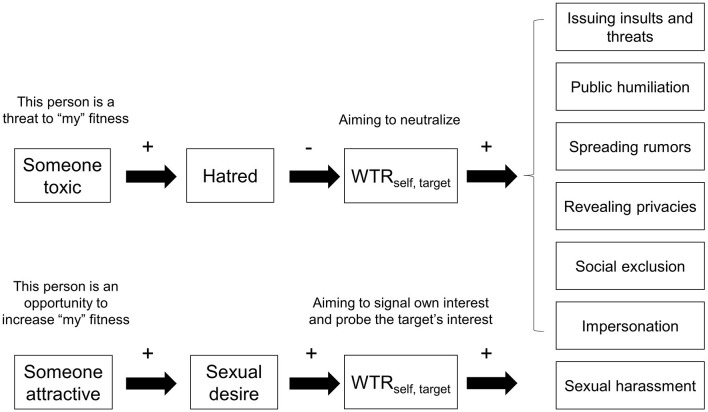
The schematic representation of the predictions made by the neutralization hypothesis of cyberaggression. WTR_self, target_ = the welfare tradeoff ratio respondents themselves (i.e., potential aggressors) hold against a target; the plus sign (+) reads “positively predicts”; and the negative sign (-) reads “negatively predicts”.

Our analysis suggests at least three venues for future research: (1) the role of hatred vis-à-vis anger and other emotions (e.g., disgust) in the process leading to cyberaggression, (2) the role of WTR in that process, and (3) the potentially distinctive mechanisms underlying sexual harassment and other tactics of cyberaggression. Figure 1 summarizes our argument.

As shown in Figure 1, from a prospective aggressor's perspective, detecting a toxic person would activate hatred, set a negative WTR_self, target_, and subsequently motivate cyberaggression. Delton and Robertson (2016) described an economic game that validly measures the mental computation of WTR. In the game, respondents would be asked to indicate with a series of binary choices whether they are willing to forgo certain monetary amounts to have another person gain or lose some money. With this method, we will be able to capture how respondents (i.e., the potential aggressors) perceive WTR_target, self_ and set WTR_self, target_. We can then test whether hatred, anger, and/or other emotions mediate the correlation between perceived WTR_target, self_ and WTR_target, self_ and subsequently predict the intent to cyberaggress. As for testing our conjecture on sexual harassment being a separate category of cyberaggression, extant studies (Bendixen and Kennair, 2017; Zapata-Calvente et al., 2019) provide the basic methodological framework. The key is to incorporate the measure of WTR to test whether a positive WTR_self, target_ and sexual desire (but not hatred) positively predict online sexual harassment.

## 4. Conclusion

In this article, we argued that cyberaggression is produced by a psychological system (i.e., hatred) designed by natural selection to detect and eliminate fitness threats. We do not claim that hatred is the only system that generates the intent to cyberaggress but believe that the neutralization hypothesis has the potential of providing an integrative account of the behavior in question. We are actively testing the neutralization hypothesis with the methods outlined above, and eager to share our findings with interested audiences form academia and the general public alike.

## Author contributions

JZ conceived and wrote the paper.
